# PSI-BLAST-ISS: an intermediate sequence search tool for estimation of the position-specific alignment reliability

**DOI:** 10.1186/1471-2105-6-185

**Published:** 2005-07-21

**Authors:** Mindaugas Margelevičius, Česlovas Venclovas

**Affiliations:** 1 Institute of Biotechnology, Graičiūno 8, LT-02241 Vilnius, Lithuania

## Abstract

**Background:**

Protein sequence alignments have become indispensable for virtually any evolutionary, structural or functional study involving proteins. Modern sequence search and comparison methods combined with rapidly increasing sequence data often can reliably match even distantly related proteins that share little sequence similarity. However, even highly significant matches generally may have incorrectly aligned regions. Therefore when exact residue correspondence is used to transfer biological information from one aligned sequence to another, it is critical to know which alignment regions are reliable and which may contain alignment errors.

**Results:**

PSI-BLAST-ISS is a standalone Unix-based tool designed to delineate reliable regions of sequence alignments as well as to suggest potential variants in unreliable regions. The region-specific reliability is assessed by producing multiple sequence alignments in different sequence contexts followed by the analysis of the consistency of alignment variants. The PSI-BLAST-ISS output enables the user to simultaneously analyze alignment reliability between query and multiple homologous sequences. In addition, PSI-BLAST-ISS can be used to detect distantly related homologous proteins. The software is freely available at: .

**Conclusion:**

PSI-BLAST-ISS is an effective reliability assessment tool that can be useful in applications such as comparative modelling or analysis of individual sequence regions. It favorably compares with the existing similar software both in the performance and functional features.

## Background

Protein sequence alignments are at the heart of many biological applications such as sequence database searches, annotation of new sequences, inference of functional regions, comparative protein modeling. Modern sequence comparison methods (e.g. PSI-BLAST [1]) often can reliably establish an evolutionary link between two proteins and align them even if they share little sequence similarity. However, the resulting significant match between these protein sequences may well include incorrectly aligned regions that are impossible to identify by straightforward inspection. Usually, the lower is the sequence similarity the more challenging is to distinguish alignment regions that can be trusted from those that may have errors. Yet, such a distinction is very important if the exact correspondence of residue positions in sequence alignments is used to extrapolate the biological information from one protein to another. Modeling protein structure by comparison (comparative modeling), identification of active site residues, selection of sites for point mutations are just a few examples where the reliability of aligned positions is critical.

The importance of delineating reliable alignment regions has been recognized more than a decade ago, however, earlier studies focused on pairwise alignments [2-5]. Currently, due to abundant sequence data, most protein sequence comparisons are performed within the context of multiple homologs, and the importance of pairwise alignments has diminished. By including multiple homologous sequences, methods such as PSI-BLAST are able to reliably detect more distant evolutionary links and also produce more accurate alignments. Unfortunately, even most advanced sequence alignment methods do make mistakes and the identification of reliable alignment regions remains an important problem. Estimation of position-specific alignment reliability is being addressed in some recent multiple sequence alignment methods [e.g. [6,7]]. However, in the multiple alignment case the position-specific reliability index estimates the overall proportion of correct pairwise matches in each alignment column without specifying the contribution of individual sequences. Yet in applications such as comparative modeling usually it is more important to know the position-specific alignment reliability for a given sequence pair than for the whole set of aligned sequences. Recently, a growing understanding of the importance of the problem led to several studies aiming at identification of reliable alignment regions for a pair of sequences within the context of multiple homologs. For example, one of these studies found that a substantial number of misaligned positions could be removed using the near-optimal alignment information [8]. Two other recent methods have been developed that predict reliable alignment regions either directly from a generated sequence profile [9,10] or using a consensus result of several alignment algorithms [11,12]. Both latter methods are implemented as web-based servers, which makes them easily accessible and simple to use, but not without certain limitations. For example, both servers require that one of the two sequences in the alignment would have a corresponding PDB structure, which in turn would have to be present in local databases used by these servers.

Here, we present the PSI-BLAST Intermediate Sequence Search tool (PSI-BLAST-ISS) that is primarily designed to help identify reliable regions of the alignment as well as suggest potential alignment variants in unreliable regions. In comparative modeling PSI-BLAST-ISS can also help identify best matching structural templates. In addition, PSI-BLAST-ISS can be used to detect remote homologs that cannot be identified by a straightforward single PSI-BLAST search. However, it should be noted that the detection of remote homologs, unlike in the original and subsequent implementations of the Intermediate Sequence Search (ISS) strategy [13-17], is not the main purpose of our tool.

Since PSI-BLAST-ISS might be most useful in comparative modeling we are going to refer to the sequence pair of interest as the target (query) and the template (reference) sequences throughout the article. However, it should be emphasized that the tool can be applied for any protein sequences that could be linked through common homologs, independently whether the three-dimensional structure for any of them is available or not.

The main idea of PSI-BLAST-ISS is to obtain a number of alignment variants for the sequence pair of interest (target and template) and analyze their consistency. This idea has stemmed from previous manual analysis of multiple PSI-BLAST alignment variants suggesting that regions where variants do agree are likely to be aligned correctly and display close structural similarity [18].

## Implementation

**Figure 1:**
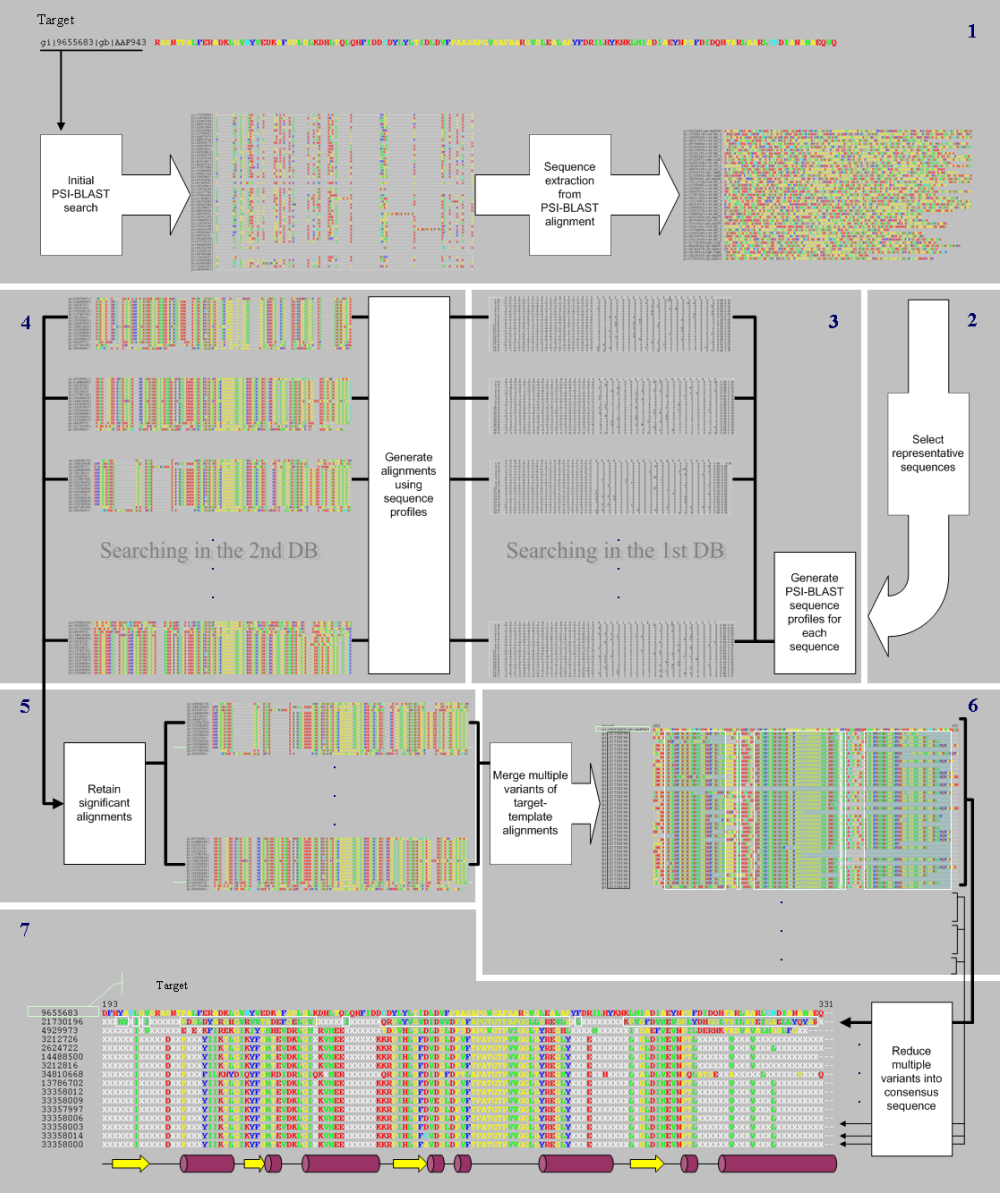
**Main steps in the PSI-BLAST-ISS execution**. PSI-BLAST-ISS comprises seven main steps to produce consensus sequence alignment starting with the target sequence. The position-specific alignment reliability can be estimated either from individual target-template multiple alignments obtained in step 6 or from the combined alignment of consensus template sequences (step 7). For example, in this figure (step 7) only two template sequences are estimated to be reliably aligned with the last helix of the target, other templates lack consensus in this region.

The whole PSI-BLAST-ISS procedure may be described as the following steps: (1) identification of multiple sequences related both to the target and template sequences, (2) formation of a representative set from these sequences by filtering out close homologs, (3) generation of sequence profiles for each sequence from this representative set by searching a sequence database with PSI-BLAST, (4) using each of the generated profiles to search a second sequence database that includes sequences of both the target and the template, (5) retention of all the instances of significant matches between the target and the template, (6) merging all significant target-template alignments by taking the target sequence as a frame of reference and (7) reducing the multiple variants of aligned template into the consensus sequence. The latter option enables contrasting of the region-specific reliability for multiple target-template alignments simultaneously. All the seven main steps are illustrated in a sketch of the data flow (Fig. 1) and are described in more detail below.

As an input, PSI-BLAST-ISS takes the target sequence in FASTA format and a file containing a number of parameters that enable a user both to specify sequence databases and to control the execution of the whole ISS procedure at every step. The target sequence is initially searched against a sequence database to collect intermediate sequences (step 1). By default, the target is searched against the non-redundant sequence database. Intermediate sequences are collected from the user-specified PSI-BLAST iteration in the resulting output file using the expectation value (E-value) threshold provided as a parameter. The reduced representative sequence set is constructed by filtering the initial set to a user-defined percentage of sequence similarity with CD-HIT (Li et al., 2001), the sequence clusterization program (step 2). Optionally, a user may introduce a strict limit to the number of sequences to be included in the representative set or even supply independently pre-selected set of sequences. A PSI-BLAST-ISS user can also choose whether to collect intermediate sequences as complete protein sequences or just as sequence fragments matching the target sequence. In the case when the target sequence represents a domain that is also found in multidomain proteins the ability to select only homologous fragments of matching sequences may help to keep the ISS procedure from straying into the realm of unrelated sequences. Each of the intermediate sequences is used to generate a sequence profile in the form of the PSI-BLAST checkpoint file by running a user-defined number of PSI-BLAST iterations (step 3). The resulting checkpoint files are then used to restart PSI-BLAST searches in a second sequence database specified by the user (step 4). This database is expected to include sequences of both proteins of interest (target and template). In a common situation, when the template represents a structural template intended for use in comparative modeling, such a database may be derived by simply appending the target sequence to the PDB sequence database. In this case there is no need to define template(s) in advance since they are identified automatically. Searches against the second database generate corresponding multiple sequence alignments that contain a number of target-template alignment variants. The significance of the target-template alignment is then determined by counting the number of alignment variants that satisfy the expectation value threshold (step 5). Both parameters can be specified by the user. The significant target-template alignment variants are extracted and merged into a single multiple sequence alignment, where the target sequence is aligned with multiple instances of the template sequence according to different alignment variants (step 6). Such an alignment immediately reveals the regions where most (or all) alignment variants are identical and thus might be considered reliable as well as those regions where there is little agreement between alignment variants and therefore unreliable. Often it is useful to analyze position-specific reliability for target alignments with multiple templates. However, it may be inconvenient to contrast/compare at once many multiple sequence alignments obtained by PSI-BLAST-ISS. To make this task easier we introduced a step (step 7) that reduces template alignment variants into a consensus template sequence for each of the target-template alignments. The consensus sequence is generated by analyzing each column of the alignment. A residue is considered conserved in the consensus template sequence if its repetition count in the corresponding position exceeds the user-defined conservation threshold.

PSI-BLAST-ISS currently is implemented as a standalone UNIX-based tool meant to be installed and run locally. It consists of fairly independent modules linked together using Perl. Some of the sequence data processing tasks in PSI-BLAST-ISS are handled by a few modified SEALS scripts [19].

## Results and Discussion

### PSI-BLAST-ISS output

PSI-BLAST-ISS produces several types of results. Perhaps the most informative output file is the FASTA-formatted sequence alignment between the target and automatically detected multiple template sequences, each represented as a consensus sequence derived from multiple alignment variants. The definition line for each consensus template sequence indicates the strength of the consensus in the interval from 0 to 1 (0 – no consensus, 1 – complete agreement) and the number of significant target-template alignment variants that were used to produce the consensus. This output provides a possibility to simultaneously assess the alignment reliability between the target and multiple templates in a region-specific manner. In addition, the consensus strength and the number of significant target-template alignments may help in selecting templates that are structurally most consistent with the target. PSI-BLAST-ISS also produces individual FASTA-formatted multiple sequence alignment files for each target-template pair, where the target is aligned with multiple copies of the same template according to obtained multiple alignment variants. These alignment files provide a visual assessment of the region-specific alignment reliability as well as candidate alignment variants if further analysis of unreliably aligned stretches is needed. Finally, all the template sequences represented in the consensus alignment are collected together in a separate output file.

### Performance of PSI-BLAST-ISS in the assessment of alignment reliability

Like for any method it is important to know how PSI-BLAST-ISS performs relative to other available methods. At the time of this study we have been aware of only two publicly available servers that estimate the position-specific reliability of sequence alignment using information from multiple sequences: the Consensus server [12] and SQUARE [9]. Of those, the performance of PSI-BLAST-ISS could be directly compared only with the Consensus server since SQUARE estimates reliability only for the supplied alignment and does not address the problem of alignment itself.

To compare PSI-BLAST-ISS and Consensus we chose protein sequences provided as prediction targets in the last round [20] of the community-wide protein structure prediction experiment known as CASP . These proteins represent a variety of different structural folds and different degree of similarity to known structures. We ran PSI-BLAST-ISS for all the target sequences assessed in CASP6, but only those, for which PSI-BLAST-ISS with default parameters generated at least ten significant alignment variants with a structural template, were further analysed. The "gold standard" in evaluating sequence alignments is to compare them with the alignments derived from protein structure superposition. For most targets PSI-BLAST-ISS detected multiple templates but for evaluating its performance we only considered a single template for each target. The DaliLite structure comparison program [21] was used both to select the template structurally closest to the target (the highest DaliLite Z-score) and to derive the "gold standard" alignment between the target and the template. The performance of PSI-BLAST-ISS was then assessed by checking to what extent alignment regions considered by PSI-BLAST-ISS to be reliable (consensus sequence assigned) agree with DaliLite structure-based alignments. In parallel, the same target-template sequence pairs were submitted to the Consensus server. The regions deemed by Consensus both structurally conserved and confidently aligned (indicated with 'S') were in turn contrasted with DaliLite structural alignments. Results obtained by PSI-BLAST-ISS and the Consensus server are presented in Table 1. In the case of PSI-BLAST-ISS, results for two consensus assignment thresholds (0.8 and 0.9) are provided.

**Table 1:** Comparison of PSI-BLAST-ISS and the Consensus server performance

*Target*	*Template*	*Align length*	*Rmsd*, Å	*Seq id*, %	Consensus server		PSI-BLAST-ISS *(consensus, 0.8)*		PSI-BLAST-ISS *(consensus, 0.9)*	
					*discrepancies*	*d-len/cons-len*	*discrepancies*	*d-len/cons-len*	*discrepancies*	*d-len/cons-len*
T0196	1jny	80	1.6	33	68–77	10/62	68–74, 77	8/56	69–72	4/50
T0200	1ush	210	2.7	16	71	1/45	-	0/58	-	0/50
T0202	1u0r	245	2.0	26	59–62, 94	5/146	59, 108	2/186	-	0/144
T0204	1gup	280	2.0	25	164	1/173	139, 141	2/17	-	0/14
T0208	1i60	254	3.1	11	258–262	5/102	292	1/90	-	0/85
T0211	1eut	126	1.7	22	-	0/13	-	0/65	-	0/55
T0216	1vpb	417	2.5	25	-	0/177	38–39, 71	3/238	-	0/107
T0222	1rzm	239	2.7	14	154	1/96	154, 246, 281–285	7/143	154, 246, 281–285	7/116
T0223	1vfr	123	2.5	11	-	0/22	113–119, 121–124, 128–132	16/39	113–116	4/24
T0228	1qpn	145	3.1	11	-	0/21	170–172, 174–181	11/57	-	0/39
T0229	1ml8	125	1.9	35	120–127	8/61	82–83, 120–127	10/114	120–127	8/84
T0231	1v6f	136	1.4	79	-	0/133	-	0/130	-	0/125
T0232	11gs	199	2.1	19	-	0/32	5–6, 40, 42–43, 66, 158	7/114	6, 40, 42–43, 66, 158	6/97
T0233	1kgz	319	1.8	36	136, 325–326	3/276	136, 245, 325–327	5/306	136, 245	2/279
T0234	1g76	118	2.8	14	16	1/59	11, 13–14, 16	4/67	11, 13–14, 16	4/58
T0235	1nb8	276	2.4	26	442–443, 478	3/44	207–208, 443, 478–479	5/104	207–208, 443	3/93
T0240	1lr0	70	2.5	17	25,33	2/39	10–11, 20–22, 24–26, 28–30, 65–66	13/51	20–22, 24–26	6/43
T0244	1iim	242	2.6	24	206, 229–234	7/157	231–234	4/153	-	0/115
T0246	1cnz	353	1.4	57	-	0/315	-	0/313	-	0/276
T0247	1pj5	338	2.1	25	76, 115–126, 128–129, 162–165, 261, 302	21/227	76, 162, 305	3/227	76	1/150
T0264	1vhv	244	2.1	34	60	1/120	17–19	3/92	17–19	3/81
T0265	1sfx	87	3.0	25	-	0/50	-	0/50	-	0/42
T0266	1dbu	145	1.8	25	60–65, 77	7/91	28, 77	2/132	-	0/95
T0267	1tiq	165	2.1	16	80–83	4/77	68, 80–81	3/102	68, 80–81	3/91
T0268	1m6y	277	1.6	49	121, 153–154	3/216	44, 126, 249	3/258	126	1/216
T0269	1qmv	182	2.1	35	89–92, 95, 119–120	7/125	119–120	2/128	-	0/99
T0274	1i0r	144	1.7	24	43,102–103	3/86	8, 43, 73	3/107	43	1/87
T0275	1mjh	125	2.0	30	26–30, 41–45	10/75	26–30, 41–42	7/107	29–30, 41	3/92
T0276	1sbq	153	1.7	26	92–94	3/56	18, 39, 150–151	4/122	-	0/84
T0279	1jr2	240	5.9	16	-	0/31	144–145, 202–204, 258	6/68	-	0/6
T0280	1o5o	144	3.0	19	170–175	6/55	119	1/51	-	0/46
T0282	1gq6	275	2.4	21	245	1/129	41–42, 208–210	5/202	42	1/138
							
					*Total:*	113/3311	*Total:*	140/3947	*Total:*	57/3081
					*Average per target:*	3.5/103.5	*Average per target:*	4.4/123.3	*Average per target:*	1.8/96.3
					*Fraction*	3.4%	*Fraction*	3.5%	*Fraction*	1.9%

Target-template structure-based alignments that were used as reference are characterized by the number of superimposed residues (column *Align length*), root-mean-square deviation of their Cα atoms (*Rmsd*), and the sequence identity (*Seq id*). Differences between each structure-based alignment and alignments obtained either by the Consensus server or PSI-BLAST-ISS are reported in corresponding *discrepancies *columns. The discrepancies are reported as segments, and their begin-end positions are given with respect to the target sequence. Only consensus segments of at least 3 residues were considered. Columns *d-len/cons-len *provide ratios between the length of discrepancies (*d-len*) and the total length of the alignment considered to be reliable (*cons-len*) by the corresponding method.

The data in Table 1 indicate that using consensus assignment threshold of 0.8 PSI-BLAST-ISS produces more extensive coverage than the Consensus server at a slightly higher rate of discrepancies with DaliLite structure-based alignments. The visual inspection of the superimposed structures revealed that most of these alignment discrepancies are minor. Some of them occur simply due to a difference in a gap placement position when, for example, one of the structures in the pair has either single residue insertion or deletion. Some other discrepancies are short stretches at the transition of a conserved secondary structure into a non-conserved loop and also can hardly be considered alignment errors. Most of these minor discrepancies disappear once the consensus assignment stringency is increased to 0.9. While the coverage becomes only slightly less extensive than for the Consensus server, the discrepancy rate is almost two times lower. Thus the increase in the stringency of the PSI-BLAST-ISS consensus assignment lowers the chances of including both non-conserved structural motifs and alignment errors within regions assigned as reliable.

### Utility of multiple alignment variants

**Figure 2:**
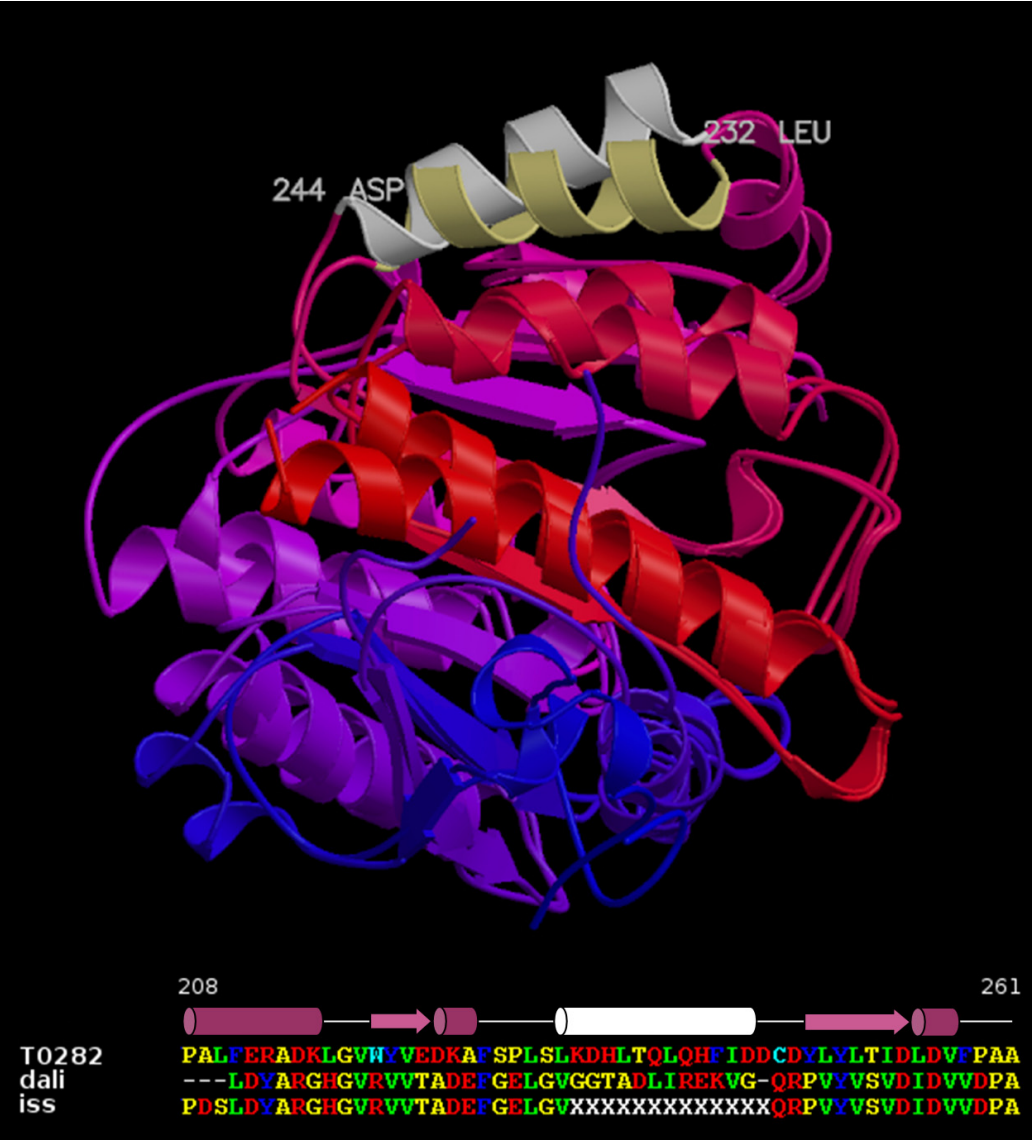
**Lack of the alignment consensus may reflect a structural divergence of the motif**. One of the α-helices (light color) displays a considerable difference in orientation in the two superimposed structures, target T0282 and the template 1gq6. All other regions of the structures are assigned the color gradient ranging from blue (N-termini) to red (C-termini). The lower part of the figure shows this α-helix and adjacent regions of T0282 aligned with 1gq6 according to both structural correspondence (dali) and a consensus alignment produced by PSI-BLAST-ISS (iss). The secondary structure of the target T0282 is shown above the sequence alignment.

**Figure 3:**
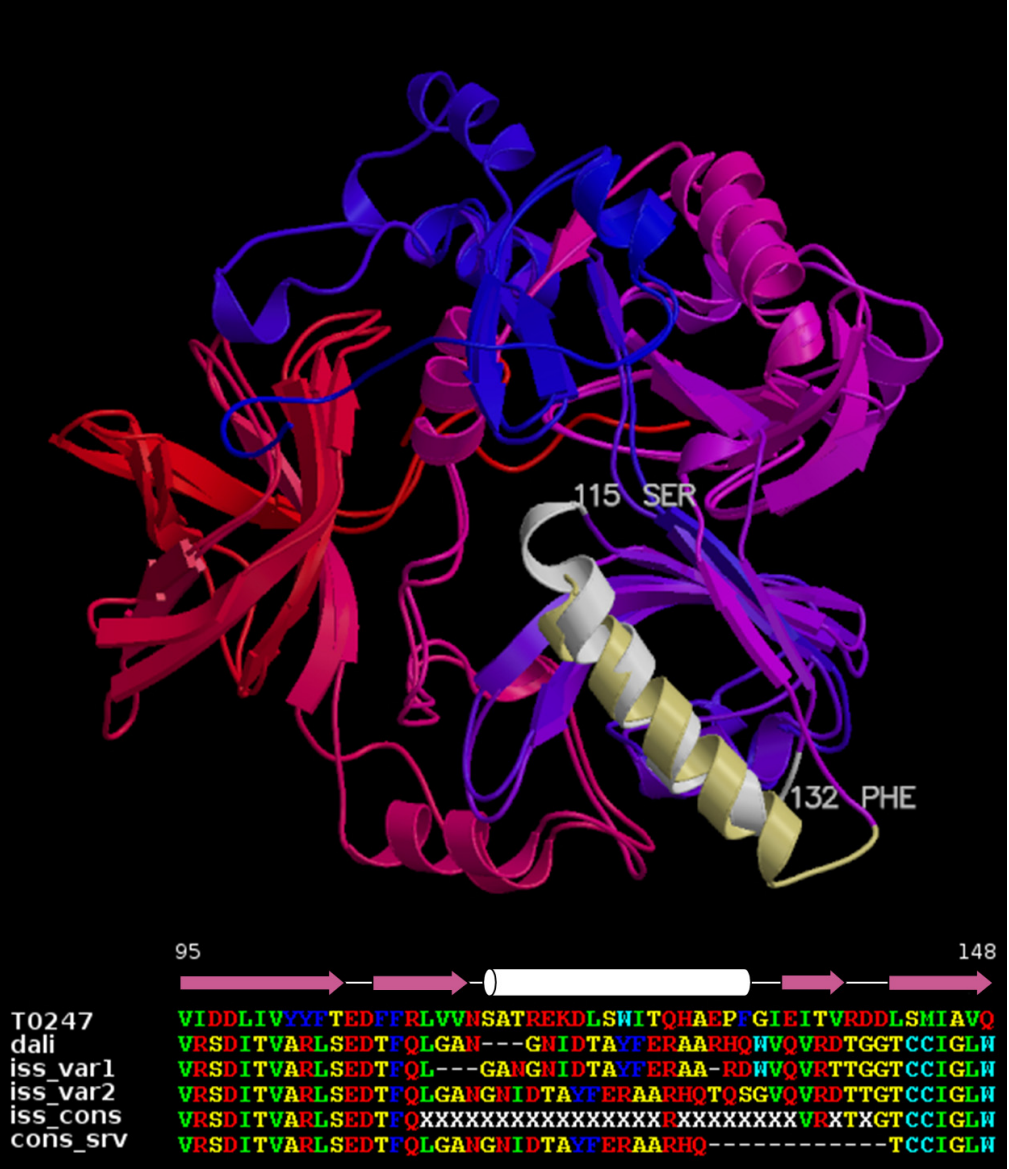
**Lack of the alignment consensus in a structurally conserved region due to variable adjacent regions**. Structural superposition of T0247 with the template 1pj5. The considered T0247 α-helix (white) superimposes fairly closely with the corresponding α-helix (light yellow) in 1pj5, but has an insertion at one end and a deletion at the other end. The lower part of the figure shows the α-helix and adjacent regions of T0247 aligned with the corresponding fragment of the 1pj5 sequence. For the 1pj5 sequence the structure-based alignment (dali), the PSI-BLAST-ISS consensus alignment (iss), two individual PSI-BLAST-ISS alignment variants (iss_var1 and iss_var2) and the Consensus server alignment (cons_srv) are shown. The alignment obtained by the Consensus server includes only residues considered to be aligned confidently (residues assigned to 'S'). The secondary structure diagram for T0247 is also shown above the sequence alignment.

A useful feature of PSI-BLAST-ISS is that it provides multiple alignment variants between the target and each template. Results in Table 1 show that regions where most alignment variants agree (consensus 0.8 or higher) usually represent reliably aligned structurally conserved stretches of protein chain. In contrast, the absence of a strong PSI-BLAST-ISS consensus indicates that any alignment variant in the corresponding region is not to be trusted. The unreliable alignment may point to a structural difference in the region such as in an example shown in Figure 2. Another possibility is that the structure of the region is conserved, however, because of the sequence dissimilarity or the variability of adjacent regions (insertions/deletions) sequence comparison programs fail to consistently come up with the same alignment variant. If related protein structures suggest that the considered region is indeed structurally conserved the correct alignment might be present among the variants generated by PSI-BLAST-ISS. For example, in the case of T0247 (Fig. 3), PSI-BLAST-ISS did not consider one of the structurally fairly conserved α-helices (115–132) reliably aligned with the corresponding region of the structural template (1pj5) and did not assign the consensus. Nevertheless, PSI-BLAST-ISS did suggest the correct alignment as one of the two major variants. In contrast, the Consensus server did supply a confident yet wrong alignment. It is easy to see that in this particular case an insertion on one side and a deletion on the other side of the otherwise conserved α-helix present a formidable problem for sequence-based methods. On the other hand, in cases like this, it might be possible to make a confident selection of the correct alignment by applying other methods that go beyond sequence comparison. In the homology modeling an assessment of different alignment variants within the context of the three-dimensional structure might be one of the potential solutions [e.g. [22]].

### Selection of representative templates (homologs)

Often there is a need to choose a single or just a few best templates from a large number of distantly related target homologs. This becomes a challenge at low sequence similarity when the sequence signal is no longer a good indicator of structural relatedness (for example, see Fig. 1 in [23]). The number of significant target-template variants retained by PSI-BLAST-ISS for generation of consensus template sequence might guide such selection of the template(s). The higher is the number of target-template alignment variants that are accepted as significant, the closer structural relationship between them might be expected. This number is directly available from the file containing the alignment between the target and the individual template and is also reported within the definition line for each template in the consensus alignment file.

### Detection of distant evolutionary relationships (homologous folds)

Multiple initiation points in the PSI-BLAST-ISS procedure ensure that the space of homologous sequences is explored more exhaustively than in the case of a single query-based search. Owing to that, PSI-BLAST-ISS may uncover distant evolutionary relationships, which are not seen if only a single query-initiated PSI-BLAST search is performed. In other words, PSI-BLAST-ISS may serve as a transitive PSI-BLAST tool for the detection of homologous folds. To test this PSI-BLAST-ISS capability we used CASP6 Homologous Fold Recognition targets (FR/H). These targets do have evolutionary related structures in the PDB database but these relationships could not be detected by PSI-BLAST searches initiated with the target sequence. For this test we required at least one significant match to a PDB structure (template) from all intermediate sequence searches. To make the comparison compatible with the CASP6 setting we only considered structural templates that were available from PDB at the time of the CASP6 experiment. We also excluded from consideration those FR/H CASP6 targets, for which at least one domain could be matched to a PDB structure using a straightforward PSI-BLAST search. As a result, out of fourteen considered FR/H targets, PSI-BLAST-ISS was able to identify related structures for four of them (1rxx for T0203, 1pk6 and several others for T0206, 1jx7 for T0224, 1qpn and other structures for T0228). An interesting case is T0228. While direct PSI-BLAST search initiated with the T0228 sequence failed to find any related structure, PSI-BLAST-ISS identified several structures producing over ten significant matches each (a default parameter). The latter result stresses the fact that sometimes the space of homologous sequences might be skewed in such a manner that a single sequence search may not be very effective in identifying important relationships.

## Conclusion

We have described PSI-BLAST-ISS, a tool for delineating reliable alignment regions and suggesting possible alignment choices in unreliable yet structurally conserved regions. PSI-BLAST-ISS might be most useful in assessing target-template alignments in comparative modeling or judging whether the interpolation of biological information directly form alignments is feasible for individual sequence regions. Unlike two other recently published methods for predicting reliable alignment regions (SQUARE and the Consensus server) PSI-BLAST-ISS is not confined to reference (template) sequences with known three-dimensional structure. The performance of PSI-BLAST-ISS in alignment reliability estimation was directly compared with the Consensus server. We find that on a set of CASP6 targets PSI-BLAST-ISS on average is able to produce more extensive coverage of confident alignment or fewer errors, depending on the selected consensus stringency. The functionality of PSI-BLAST-ISS also extends into detection of non-apparent distant homologous relationships.

## Availability and requirements

**Project name: **The PSI-BLAST intermediate sequence search tool (PSI-BLAST-ISS)

**Project home page: **

**Operating systems: **Unix-based platforms

**Programming language: **Perl

**Other requirements: **locally installed PSI-BLAST and CD-HIT (optional)

**License: **None

**Any restriction to use by non-academics: **None

## Authors' contributions

MM carried out the software development, programming work and participated in manuscript preparation. ČV conceived of the study, participated in its design and coordination and drafted the manuscript. All authors read and approved the final manuscript.
